# Electrocatalytic Reduction of Carbon Dioxide to Carbon Monoxide by a Polymerized Film of an Alkynyl-Substituted Rhenium(I) Complex

**DOI:** 10.1002/cctc.201200904

**Published:** 2013-04-12

**Authors:** Engelbert Portenkirchner, Jacek Gasiorowski, Kerstin Oppelt, Stefanie Schlager, Clemens Schwarzinger, Helmut Neugebauer, Günther Knör, Niyazi Serdar Sariciftci

**Affiliations:** [a] Linz Institute for Organic Solar Cells (LIOS), Institute of Physical Chemistry, Johannes Kepler University Linz (Austria)Fax: (+43) 732-2468-8770 E-mail: Engelbert.Portenkirchner@jku.at; [b] Institute of Inorganic Chemistry, Center for Nanobionics and Photochemical Sciences (CNPS), Johannes Kepler University Linz (Austria); [c] Institute for Chemical Technology of Organic Materials, Johannes Kepler University Linz (Austria)

**Keywords:** electrochemistry, heterogeneous catalysis, polymerization, reduction, rhenium

## Abstract

The alkynyl-substituted Re^I^ complex [Re(5,5′-bisphenylethynyl-2,2′-bipyridyl)(CO)_3_Cl] was immobilized by electropolymerization onto a Pt-plate electrode. The polymerized film exhibited electrocatalytic activity for the reduction of CO_2_ to CO. Cyclic voltammetry studies and bulk controlled-potential electrolysis experiments were performed by using a CO_2_-saturated acetonitrile solution. The CO_2_ reduction, determined by cyclic voltammetry, occurs at approximately −1150 mV versus the normal hydrogen electrode (NHE). Quantitative analysis by GC and IR spectroscopy was used to determine a Faradaic efficiency of approximately 33 % for the formation of CO. Both values of the modified electrode were compared to the performance of the homogenous monomer [Re(5,5′-bisphenylethynyl-2,2′-bipyridyl)(CO)_3_Cl] in acetonitrile. The polymer formation and its properties were studied by using SEM, AFM, and attenuated total reflectance (ATR) FTIR and UV/Vis spectroscopy.

## Introduction

The recycling of CO_2_ by electrocatalytic conversion to gaseous or liquid fuels that use renewable energy is a promising pathway towards a carbon-neutral fuel cycle. The reduction of CO_2_ usually requires a high negative potential of nearly 1.9 V versus the normal hydrogen electrode (NHE) for a one-electron reduction.^1^ The actual redox potential, however, is much higher than the Nernst potential owing to barrier-induced overpotentials. To decrease the actual redox potential, other pathways that involve a multielectron process are necessary, which require suitable catalysts.^2^–^5^

Re compounds with bipyridine (bipy) ligands demonstrate excellent properties in terms of activities and lifetimes for the selective homogeneous CO_2_ reduction to CO.^6^, ^7^ In particular, [Re(2,2′-bipyridyl)(CO)_3_Cl], which was characterized in this context for the first time by Hawecker et al. in 1984, was found to show high Faradaic efficiencies and no significant decrease in performance because of catalyst degradation over several hours was observed.^8^, ^9^ Furthermore, Ley and Schanze have studied the excited-state properties of several different Re bipy complexes in great detail.^10^ The catalytic effects of these compounds have already been further improved.^9^, ^11^

**Scheme 1:**
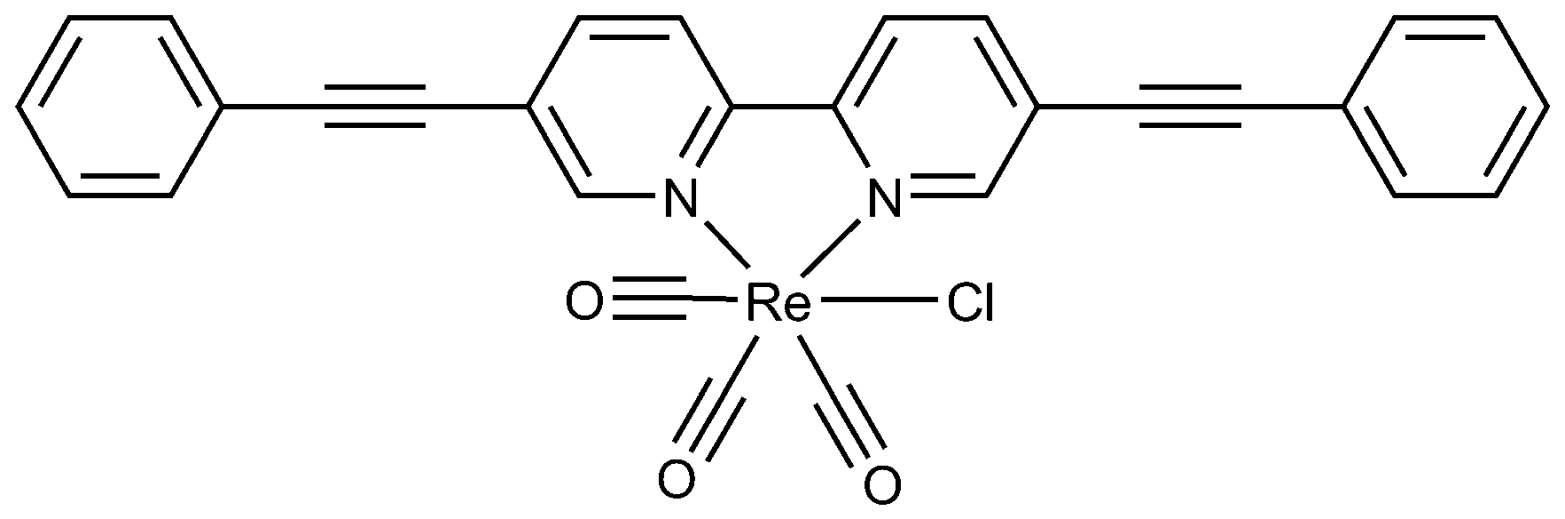
Structure of [Re(5,5′-bisphenylethynyl-2,2′-bipyridyl)(CO)_3_Cl] (**1**).

Recently, we reported the homogenous electro- and photocatalytic reduction of CO_2_ to CO by using the alkynyl-substituted Re^I^ complex [Re(5,5′-bisphenylethynyl-2,2′-bipyridyl)(CO)_3_Cl] (**1**) (Scheme 1)^12^ as well as its synthesis, structure, photophysics, and spectroscopic characterization.^13^ Compound **1** showed a lower reduction potential and higher rate constant for the reduction of CO_2_ to CO than Re-based catalysts reported previously.

Although homogeneous catalysis is easier to characterize and mechanistically better understood than heterogeneous catalysis, it has several disadvantages. Large amounts of expensive catalyst are necessary for efficient CO_2_ reduction, and the system is limited by the solubilities of the active species, which often allow the use of only a small variety of solvents that do not necessarily match the desired high CO_2_ solubility properties. Furthermore, homogeneous catalysts may sometimes face solution deactivation pathways, for example, the dimer formation of Re compounds with bipy ligands in nonaqueous solution systems.^14^ One way to overcome these problems is to immobilize the catalyst on an electrode and thereby change from homogeneous to heterogeneous catalysis. Therefore, the aim of this work is to immobilize **1** onto an electrode and determine its potential for heterogeneous catalysis towards CO_2_ reduction.

In the past, the most frequently reported ways to immobilize Re catalysts onto solid electrodes were either the insertion of the molecule into a polymer matrix^15^–^17^ or the chemical modification of the ligand with a functional group that allowed polymerization to form a redox polymer.^18^–^20^

In this work, we report the electrocatalytic reduction of CO_2_ to CO by using a polymerized film of monomer **1** (**2**). The film growth on a Pt working electrode was performed by potentiodynamic scanning in a N_2_-saturated acetonitrile solution that contained TBAPF_6_ (0.1 m; TBA=tetrabutylammonium) and catalyst monomer (2 mm). The films were electrochemically characterized by using cyclic voltammetry. The catalytic properties for CO_2_ reduction were studied by using cyclic voltammetry in a CO_2_-saturated acetonitrile solution that contained TBAPF_6_ (0.1 m).

The polymerized film electrode demonstrates a high selectivity for CO_2_ reduction to CO at low reduction potentials and high current densities in acetonitrile solution.^21^, ^22^ Film **2** formed during potentiodynamic scanning was further characterized by using attenuated total reflectance FTIR (ATR-FTIR) spectroscopy to confirm the polymer growth. The optical absorption of the layer was measured and compared with that of **1**. Finally, the morphology of the film was studied by using SEM and AFM.

## Results and Discussion

### Electrochemical studies

**Figure 1:**
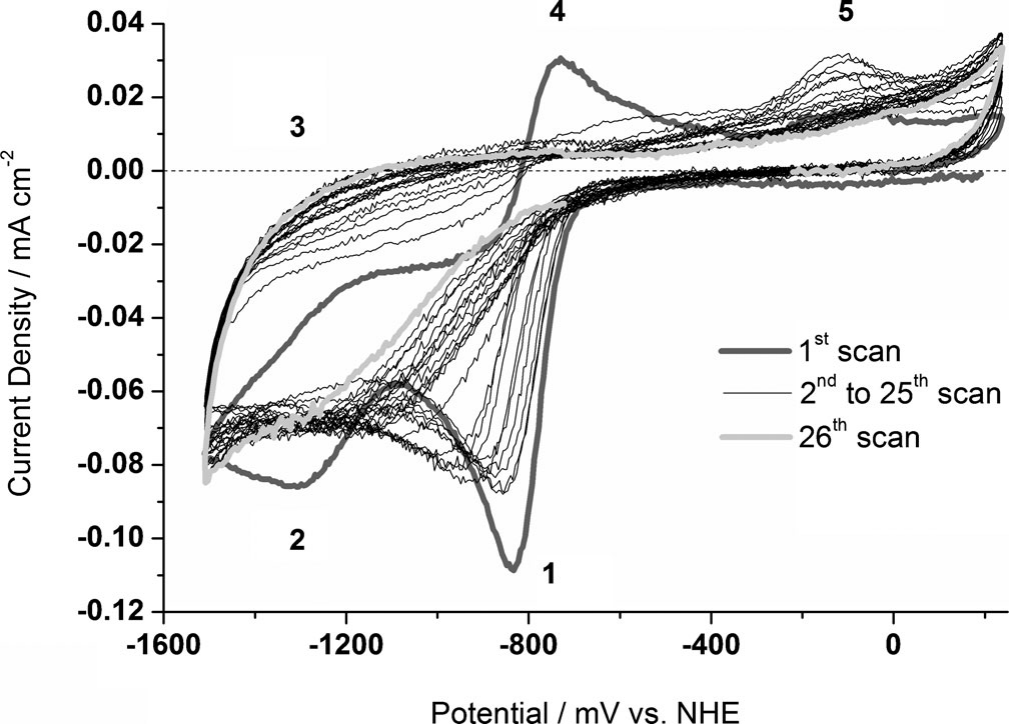
Potentiodynamic formation of rhenium catalyst film **2** on Pt from a catalyst monomer solution of **1**. First scan (thick dark grey solid line) and last scan (thick light grey solid line). Voltammograms are recorded at 50 mV s^−1^ in nitrogen-saturated acetonitrile solution containing 0.1 M TBAPF_6_ and a monomer catalyst concentration of 2 mM.

The potentiodynamic formation of **2** by the electropolymerization of **1** onto a Pt working electrode (WE) from a solution of **1** (2 mm) is shown in Figure 1.

Changes in the voltammogram with the increasing number of cycles are indicated by 1–5 in Figure 1. In the first scan (thick dark grey solid line), two distinct reduction waves of **1** are still visible. The peak at −850 mV can be attributed to a ligand-based reduction, and the reduction wave at −1300 mV can be assigned to a reduction at the metal center. The first reduction wave is partly reversible, and the reoxidation peak appears at −750 mV. Peaks 1 and 4, attributed to the monomer ligand, decrease in intensity with the increasing number of scans, which suggests a ligand-based polymerization of **1**. The polymerization is assumed to proceed through radical coupling between two electrogenerated radical species as reported previously for similar systems,^19^, ^21^ although alternative routes cannot be excluded owing to the presence of metal carbonyl species.^23^ Furthermore, the maximum of the first reduction wave shifts towards more negative potentials with the increasing number of scans. The oxidative peak 5 at approximately −100 mV initially appears and disappears after continuous scanning, which may be attributed to temporary dimer formation as described for similar systems.^14^ After approximately 25 cycles, the voltammogram shows no further changes and displays a distinct background current below −700 mV (thick light grey solid line). This background current is present over continuous scans and is indicated by 2 and 3. After film formation, **2** shows an intense violet color on the part of the electrode that was in contact with the monomer solution (see Figure 5 later).

**Figure 2:**
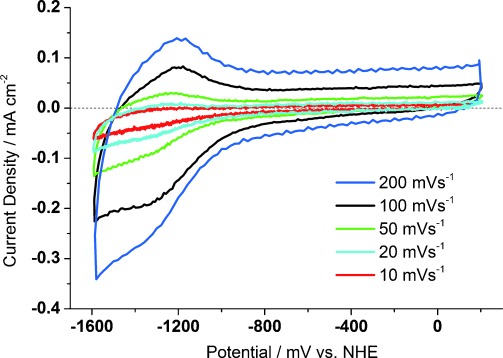
Cyclic voltammograms of **2** on Pt in N_2_-saturated acetonitrile solution that contained TBAPF_6_ (0.1 m) at different scan rates from 200 mV s^−1^ (blue solid line) to 10 mV s^−1^ (red solid line).

The electroactivity of **2** on a Pt-plate electrode at various scan rates from 200–10 mV s^−1^ is shown in Figure 2. A plot of peak current versus scan rate reveals a linear dependence, which suggests that the redox process is no longer diffusion controlled as predicted by the Randles–Sevcik equation.^24^ This further confirms the formation of an electroactive film immobilized on the Pt electrode surface (Figure S1).^25^ Additionally, the maximum reduction peak position is independent of the scan rate within the measured cycling times. This shows that the electron transfer kinetics is fast with respect to the cycling time scales, which suggests a Nernstian behavior.^26^

**Figure 3:**
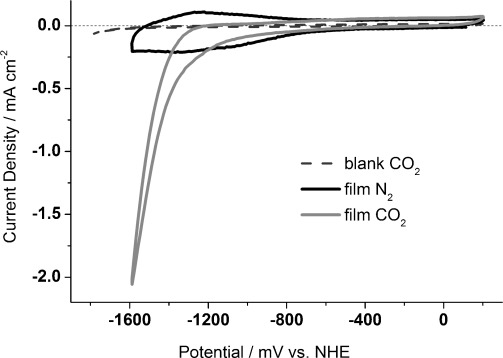
Cyclic voltammograms of **2** on a Pt-plate electrode in N_2_- (black solid line) and CO_2_-saturated electrolyte solution (solid grey line). The scan in the presence of CO_2_ shows a large current enhancement owing to the catalytic reduction of CO_2_ to CO. A scan with no catalyst film present under CO_2_ (dashed line) shows little to no reductive current. Voltammograms were recorded at 100 mV s^−1^ in acetonitrile with a Pt CE.

Cyclic voltammetry measurements of **2** on a Pt-plate electrode in N_2_- and CO_2_-saturated electrolyte solutions are shown in Figure 3. The measurement of the potential window with two Pt electrodes as the WE and counter electrode (CE) and an electrolyte solution under N_2_ shows no reductive current in the potential range from 0 to −2000 mV. If the solution is purged with CO_2_ for 10 min and no catalyst is present, a reductive current starts to flow at a potential lower than approximately −1700 mV versus NHE (dashed line).

If the Pt WE is replaced by the Pt electrode with **2** and measured in the electrolyte solution under N_2_ atmosphere, the typical reduction curve such as that shown in Figure 2 is measured again (Figure 3, black solid line). However, if the electrolyte solution is saturated with CO_2_, a high, nonreversible reductive current enhancement is observed (Figure 3, solid grey line). The reductive current begins to increase at approximately −1150 mV versus NHE and can be attributed to the reduction of CO_2_ to CO.

According to previous studies on catalytically active electrodes with Re-based catalysts, the pathway for CO_2_ reduction is similar to that of a homogeneous system. The catalytic mechanism proceeds through the coordination of a CO_2_ molecule to a Re atom, which allows the reduction of CO_2_ to CO.^14^, ^19^–^21^, ^27^ As a result, the catalytic current per area depends on the number of redox active sites per surface area.

**Figure 4:**
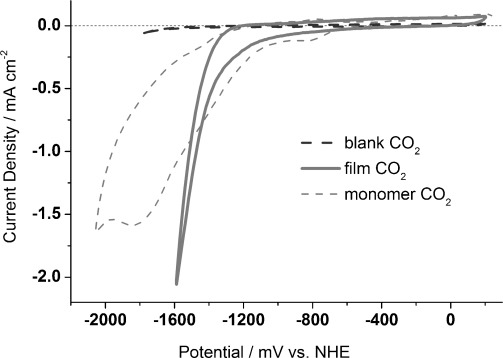
Cyclic voltammograms of **2** on a Pt-plate electrode (solid line) and a 1 mM solution of **1** (thin grey dashed line) in CO_2_-saturated electrolyte solution. The scan in the presence of CO_2_ shows large current enhancement for both systems owing to the catalytic reduction of CO_2_ to CO. Voltammograms were recorded at 100 mV s^−1^ in acetonitrile with a Pt CE. A scan with no catalyst film present under CO_2_ (thick grey dashed line) shows little to no reductive current.

A comparison between **1** in solution (thin grey dashed line) and **2** on a Pt-plate electrode (solid line) in CO_2_-saturated electrolyte is presented in Figure 4. The measurement shows that the electrochemical onset potential for CO_2_ reduction with **2** on the Pt electrode has a similar value to that of **1** in solution, which is at approximately −1150 mV versus NHE. The reductive current initially increases more rapidly for the homogeneous system with **1**. However, with increasing negative potential, the current density at **2** increases significantly faster and surpasses the reductive current of the 1 mM solution of **1** at approximately −1450 mV versus NHE.

In contrast to the cyclic voltammogram of **2** on a Pt electrode (solid line), the cyclic voltammogram of **1** in a CO_2_-saturated electrolyte solution (thin grey dashed line) still shows the quasireversible first reduction wave at approximately −850 mV. This reduction wave is attributed to the ligand of **1**. As known from previous experiments, this reductive peak does not show any current enhancement under CO_2_ saturation compared to saturation with N_2_.^12^, ^19^

CO_2_ electrolysis at a constant potential of −1600 mV versus NHE of a pure Pt-plate electrode and of **2** was performed in acetonitrile solution saturated with CO_2_. The experiment was performed in a sealed cell over 60 min. During this period, no film degradation was observed, and *I*/*t* plots are shown in Figures S2 and S3.

As a direct proof of the catalytic CO_2_ reduction capability of **2**, headspace-gas samples were withdrawn and analyzed with regard to the CO concentration by using GC and FTIR spectroscopy.

The Faradaic efficiency (*η*_F_) was calculated according to Equation (1).


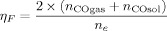
(1)

in which *n*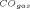 is the number of CO molecules in the gas phase, *n*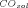 is the number of CO molecules dissolved in solution, and *n*_e_ is the number of electrons put into the system during electrolysis.

A value for *n*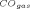 was obtained by GC and FTIR analysis, and *n*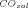 was estimated by using Henry’s Law [Eq. (2)].


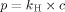
(2)

The Henry constant *k*_H_ is 2507 atm mol_solvent_ mol_CO_^−1^,^28^ *p* is the partial pressure of the solute CO, and *c* is the concentration of CO in solution. A value for *n*_e_ consumed in the CO_2_ electrolysis was determined by integration of the *I*/*t* curve over 60 min of the electrolysis experiment.

With this approach, a Faradaic efficiency for the reduction of CO_2_ to CO by **2** of approximately 33 % was calculated. The Faradaic efficiency of a 1 mM solution of **1** was previously measured by our group to be approximately 43 %.^12^ The control experiment with a pure Pt-plate electrode under otherwise identical conditions did not yield detectable amounts of CO.

It has been shown that under these conditions (CH_3_CN/TBAPF_6_), also small amounts of formate and oxalate can be formed, however, with typical Faradaic efficiencies below 1 %.^29^ Further characterization to determine the turnover number (TON) and turnover frequency (TOF) is important. These parameters will help to determine the stability and lifetime of the new catalyst film **2** and are under current investigation. Typical TONs for [Re(bipy)(CO)_3_X] (X=Cl, Br) compounds are in the order of 300.^8^, ^15^, ^30^ To determine the TON of **2**, specific information on the catalytically active Re sites on the film is necessary. However, this data is not available at present. As a first approximation, one can assume a single homogeneous active monolayer of the catalyst film on the electrode surface. This would result in a surface coverage of approximately 1.5×10^−10^ mol active Re sites per cm^2^, which is in the order of similar catalyst films.^19^ Dividing the amount of CO formed during the electrolysis experiment over 60 min by the estimated number of active Re sites results in approximately 1400 turnovers per active site and a frequency of approximately 0.4 turnovers per second. These values seem reasonable as electropolymerized Re catalysts execute approximately 30 times more turnovers per site than their monomer counterparts in solution.^19^, ^20^

### Film characterization

**Figure 5:**
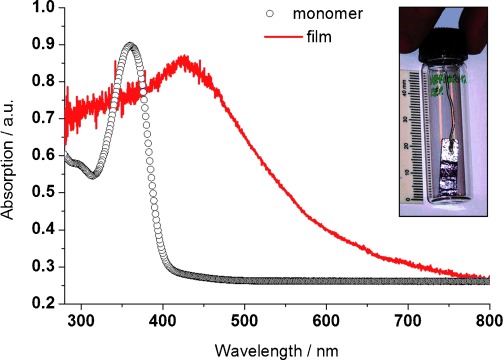
Comparison of the UV/Vis absorption spectra of **2** on a Pt-plate electrode (solid line) and of a 6.25×10^−5^ M solution of **1** in acetonitrile (298 K, 1 cm cell, black circles). A photograph of **2** electropolymerized onto a Pt-plate electrode is shown inset.

A comparison of the absorption spectra of a dilute solution of **1** in acetonitrile and of **2** on a Pt-plate electrode is shown in Figure 5.

In acetonitrile, the spectrum of **1** is dominated by an intense absorption maximum at approximately 375 nm, which results from a strong intraligand band with additional metal-to-ligand charge transfer contributions. Other weaker UV bands of intraligand origin occur between 280 and 320 nm. A detailed study on the nature of the electronic transitions and the photophysical behavior of **1** has been published previously by our group.^13^

After electropolymerization, **2** shows a redshift in the absorption band with a maximum at approximately 425 nm. Compared to that of **1**, this redshift of the absorption maximum for **2** can probably be attributed to increased delocalization of the conjugated π-electron system upon polymerization. However, the redshift is not as significant as that observed for other conjugated polymers. Therefore, the effective conjugation is probably limited to a few monomer units.

**Scheme 2:**
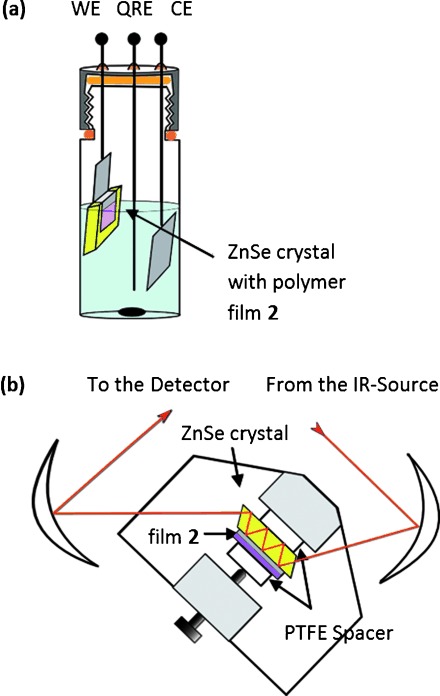
a) One-compartment cell with a ZnSe reflection element covered with a thin (10 nm) sputtered layer of Pt (gray) as the WE for the electropolymerization of **2** (violet). b) Schematic of the mounted ATR-FTIR crystal with **2** (violet) inside the spectrometer.

The polymer growth process was investigated by using an ex situ ATR-FTIR technique. For this measurement, a ZnSe reflection element covered with a thin (10 nm) sputtered film of Pt was used as the WE and a 150 nm layer of **2** was potentiostatically electropolymerized on the Pt surface of the modified ZnSe ATR crystal. The experiment was performed in a one-compartment electrochemical cell as depicted in Scheme 2 a.

**Figure 6:**
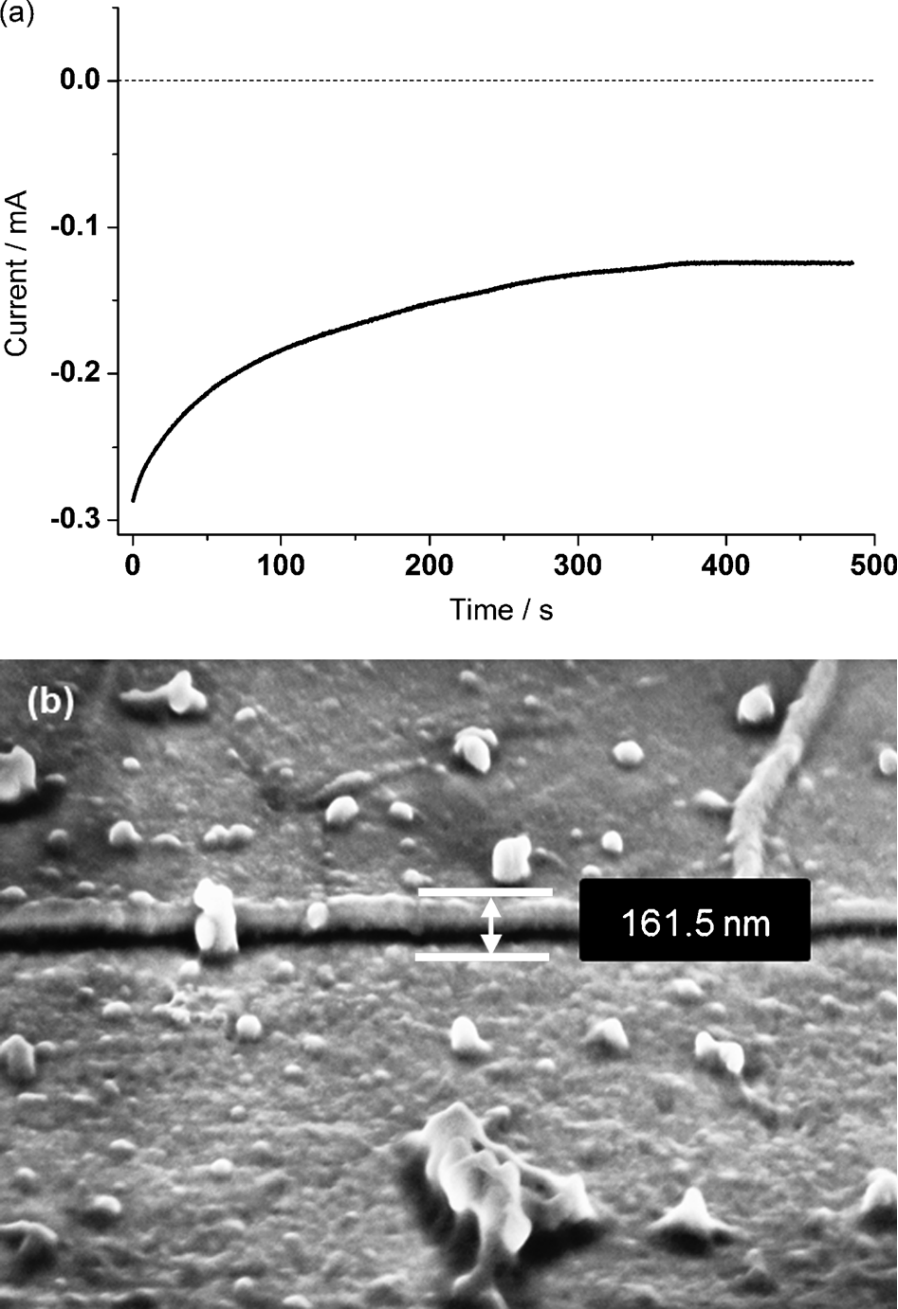
a) *I*/*t* curve for the corresponding film formation in N_2_-saturated acetonitrile solution that contained TBAPF_6_ (0.1 m) and an initial concentration of **1** of 2 mM at a constant potential of −1550 mV versus NHE. b) SEM image showing the edge of **2** on the 10 nm Pt substrate. The image was recorded at a magnification rate of 18 920 and a tilt angle of 54.0°.

The electrolyte solution contained 0.1 M TBAPF_6_ and 2 mM of **1** in acetonitrile. The electrochemical cell was connected to the potentiostat, and a constant potential of −1550 mV versus NHE was applied for 500 seconds. The electrochemical current measured as a function of time is presented in Figure 6 a. During film formation, the current dropped from −0.3 mA to approximately −0.12 mA after 400 s and stayed constant afterwards. This indicates that the film formation ceased at that time.

After electropolymerization, the ZnSe/Pt electrode with the 150 nm thick catalyst film **2** was mounted in an ATR-FTIR setup between two Teflon spacers (Scheme 2 b), and the ATR-FTIR difference absorption spectra of a pure ZnSe/Pt electrode and the ZnSe/Pt electrode with **2** were recorded.

**Figure 7:**
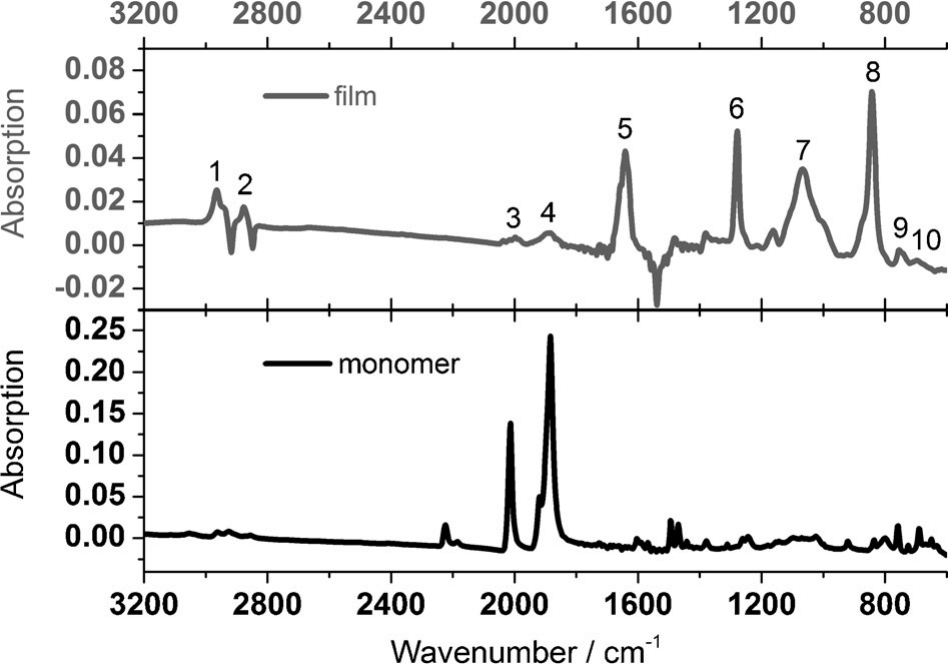
ATR-FTIR difference absorption spectra of 150 nm thick **2** on 10 nm Pt sputtered onto a ZnSe ATR crystal compared to the pure 10 nm Pt/ZnSe ATR crystal (top) and of **1** dissolved in CH_2_Cl_2_ and drop-cast onto a ZnSe ATR crystal compared to the pure ZnSe ATR crystal (bottom)

The ATR-FTIR difference absorption spectrum of the 150 nm thick film of **2** versus a pure ZnSe/Pt electrode and of **1** dissolved in dichloromethane and drop-cast onto a ZnSe ATR crystal versus the pure ZnSe ATR crystal are shown in Figure 7. In the spectrum of **2**, distinctive new peaks are observed and their positions are indicated by 1–10 in the absorption spectrum shown in Figure 7. These positions are connected with characteristic vibrations of the polymerized film. Compared to the IR spectrum of the monomer (bottom, black solid line), there is a noticeable decrease in the intensity of the monomer main peaks 3 and 4 positioned at approximately 1900 and 2000 cm^−1^, which are characteristic of C≡O vibrations.^13^, ^30^ The number of peaks and their relative intensities, however, do not change, which indicates that the incorporated catalytic centers of **1** do not decompose upon polymerization and that a facial arrangement of the carbonyl ligands is retained at the Re center. In contrast, the IR signal at 2200 cm^−1^ characteristic of C≡C vibrations almost vanishes completely, and the appearance of a new sharp peak 8 centered at approximately 848 cm^−1^ indicates the asymmetric stretching band of PF_6_^−^. It is assumed that the disappearance of the peak at 2200 cm^−1^ might be directly connected to a loss of C≡C bonds in the course of the polymerization process. The absence of solvent bands from acetonitrile at 2293, 2252, 1442, 1035, and 917 cm^−1^[31] in the spectrum suggests that the band at 848 cm^−1^ could be attributed to the presence of PF_6_^−^-containing species in the polymer phase. The significant increase of this peak could then be explained by a substitution of the Cl^−^ in **1** by PF_6_^−^ from the electrolyte during polymerization to retain charge neutrality^32^ or, because of the high relative intensity of the signal, by the uptake of a certain amount of the alkylammonium electrolyte. Peaks 1 and 2 at 2972 and 2877 cm^−1^ are attributed to the valence vibrations of additional aliphatic C–H bonds formed during polymerization or because of the presence of the alkylammonium salt. Peak 5 at 1640 cm^−1^ is characteristic of aryl-conjugated C=C bonds. The two small peaks 9 and 10 at 750 and 690 cm^−1^ are typical for out-of-plane C–H bending vibrations of monosubstituted benzene.^33^ Additionally, peak 7 at 1065 cm^−1^ might originate from poorly defined C–H vibrations along the main chain of the polymer film, which would explain the relative broadness of this absorption peak. For a better comparison of the peaks at lower wavenumbers, an expansion of the spectrum from 1800–700 cm^−1^ is presented in Figure S4.

**Scheme 3:**
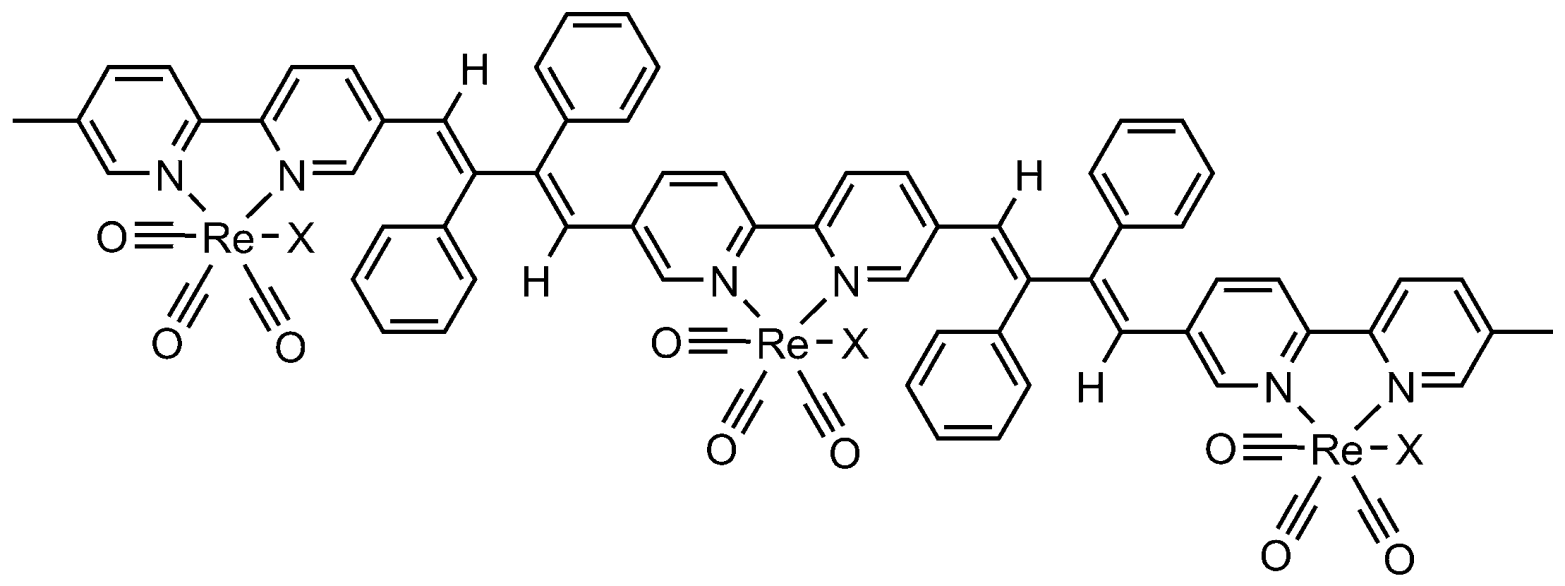
Possible substructure of the rhenium sites within the polymer film **2** in which X represents a chloride or a substituted ligand from the reaction medium.

Following this argument, we assume that polymerization occurs by radical addition similar to the mechanism published by Garcia-Canadas et al.^33^ Still, because of the strong negative potential necessary for the electropolymerization of **1**, it is likely that additional side reactions occur that account for the unassigned peak 6 at 1280 cm^−1^ in the IR spectrum shown in Figure 7. As a result, at this time it is not possible to determine the exact structure of **2**. For the catalytically active centers of **2**, however, we propose a structural motif similar to that depicted in Scheme 3.

**Figure 8:**
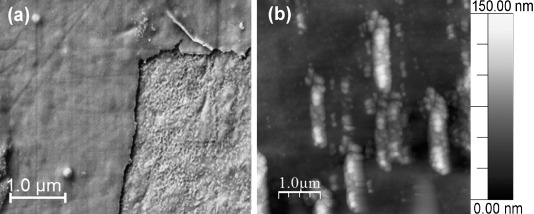
Morphology characterization of **2** by using a) SEM and b) AFM. The SEM image shows the border of an artificial scratch of 150 nm thick **2** (left side) on a 10 nm sputtered Pt substrate.

The film morphology was studied by using SEM and AFM. An SEM image of **2** formed during the potentiostatic experiment shown in Figure 6 is shown in Figure 8 a. The film surface was scratched with a spatula to see the difference between **2** (left) and the ZnSe/Pt substrate (right). A highly ordered, granular structure is observed on the right side of the image in Figure 8 a, which is characteristic of sputtered Pt. The electropolymerized film **2** (Figure 8 a, left) is significantly different from the metallic substrate. The lack of clearly visible structures on the surface of the film suggests a lack of order in the film, which is expected for polymeric films. From the tilted substrate (54.0°), a film thickness of 150 nm was measured (cf. Figure 6 b).

The roughness of the film was studied by using AFM (Figure 8 b). Close inspection of the film surface revealed the existence of particles that probably remain from the film formation process. The root mean square (RMS) of the roughness in the regions without these particles was 5.4 nm.

## Conclusions

We investigated the electrocatalytic reduction of CO_2_ to CO by using a polymerized film of [Re(5,5′-bisphenylethynyl-2,2′-bipyridyl)(CO)_3_Cl]. The CO_2_ reduction potential, determined by cyclic voltammetry, occurs at approximately −1150 mV versus NHE. Compared to that of the homogenous catalyst, the potential for CO_2_ reduction is similar. However, with increasing negative potential, the current density increases significantly faster at the catalyst film electrode **2** compared to the CO_2_ reduction with the monomeric homogeneous catalyst **1**. A Faradaic efficiency of the Re catalyst film **2** of approximately 33 % was measured for the formation of CO by using GC and FTIR spectroscopy.

The polymer formation was studied by SEM, AFM, and ATR-FTIR and UV/Vis spectroscopy. It is assumed to proceed through the coupling between two electrogenerated radical species and seems to involve a certain degree of electrolyte intercalation into the polymer phase. The Re catalyst film **2** shows a broadening in absorption with a new maximum at approximately 425 nm. The significant redshift of the absorption maximum of **2** compared to that of **1** is tentatively explained by an increase of the conjugated system upon polymerization.

The electropolymerization of **1** to form **2** is a promising way to change from homo- to heterogeneous catalysis for CO_2_ reduction, which reduces the amount of catalyst required and overcomes limitations regarding the solubility of homogeneous catalysts in general. Furthermore, the new catalyst film **2** demonstrates high selectivity for CO_2_ reduction to CO at relatively low reduction potentials and high current densities.

## Experimental Section

Unless otherwise stated, all chemicals and solvents were purchased from commercial suppliers in reagent- or technical-grade quality and used directly as received: [Re(CO)_5_Cl] (Aldrich) and TBAPF_6_ (Aldrich). ^1^H and ^13^C NMR spectra were recorded by using a Bruker Avance DPX200 NMR spectrometer.

The synthesis of [Re(5,5′-bisphenylethynyl-2,2′-bipyridyl)(CO)_3_Cl] (**1**) was performed as previously reported^13^ by the Sonogashira coupling of 5,5′-dibromo-2,2′-bipyridine with phenyl acetylene and the subsequent complexation of the obtained 5,5′-bisphenylethynyl-2,2′-bipyridine with rhenium(I) pentacarbonylchloride in toluene.^34^ The characterization of the material was performed according to the literature.^13^ ^1^H NMR (300 MHz, CD_2_Cl_2_): *δ*=9.2 (s, 2 H, bipy-H6,6′), 8.2 (s, 2 H, bipy-H3,3′), 7.7 (s, 2 H, bipy-H4,4′), 7.5 (s, 4 H, phen-H2,2′,6,6′), 7.2 ppm (m, 6 H, phen-H3,3′,4,4′,5,5′).

The electrochemical experiments were performed by using a JAISSLE Potentiostat–Galvanostat IMP 88 PC. A one-compartment cell was used that contained electrolyte solution (ca. 14 mL) and has phase (10 mL). For the controlled-potential electrolysis experiments, a Pt WE, a Pt CE, and a Ag/AgCl quasireference electrode (QRE) calibrated with ferrocene/ferrocenium (Fc/Fc^+^) as an internal reference were used. The solvent was anhydrous acetonitrile (99.8 %, Aldrich). Other than trace amounts from the supplier, no additional H_2_O was added. The half-wave potential (*E*_1/2_) for Fc/Fc^+^ was measured at 407, 433, and 403 mV versus QRE for the experiments shown in Figures 1, 2, and 3, respectively. The areas of the WEs were (0.78±0.2) and (0.66±0.2) cm^2^. To calculate the values versus the potential of the NHE, *E*_1/2_ for Fc/Fc^+^ versus NHE was taken as 640 mV.^35^

GC was conducted by using a Thermo Trace GC equipped with a thermal conductivity detector (TCD) and a Phenomenex PLTT 5A column (30 m, 0.53 mm ID, 25 μm film). The carrier gas was He (3 mL min^−1^), and the GC was programmed from 45 °C (5 min) to 300 °C (1 min) with a heating rate of 30 °C min^−1^. The injector was operated at 300 °C with a split ratio of 1:10, and the detector was operated at 200 °C with 27 mL min^− 1^ make-up gas. Samples of 1 mL were injected with a gas-tight syringe directly from the reaction vessel.

UV/Vis absorption measurements of **1** were performed in 1 cm quartz glass cuvettes at 298 K by using a Cary 3G UV/Vis spectrophotometer. The light absorption of **2** on a Pt WE was characterized by using an Ocean Optics fiber optic spectrometer and an integrating sphere for reflectance measurements (ISP-R) to measure the total integrated reflectance of surfaces. The difference in the diffuse reflectance spectra of the nontransparent electrode was compared to a white tile standard.

IR measurements were performed by using a Bruker IFS 66/S FTIR spectrometer at r.t. in ATR mode by using a mercury–cadmium telluride (MCT) detector cooled with liquid N_2_ prior to the measurements. For all ATR-FTIR measurements, a ZnSe crystal was used as the reflection element, which was cleaned by polishing with diamond paste (1 and 0.25 μm) and additionally rinsed in a reflux system with acetone. For electropolymerization, a thin (10 nm) layer of Pt was sputtered onto the ZnSe crystal, which served as a transparent WE.

To calculate the Faradaic efficiency, gas samples were withdrawn from single-compartment cells. For the FTIR gas analysis, a gas-tight transmission cell with ZnSe windows was designed to measure the IR absorption in the transmission mode.^12^

The surface morphology and film cross-sections were characterized by SEM by using an AURIGA microscope with a ZEISS 1540XB CrossBeam ultrahigh resolution GEMINI field emission column. The surface morphology of the films was characterized by using an AFM, Digital Instruments Dimension 3100, Veeco Metrology group, in the tapping mode.

## Supplementary material

As a service to our authors and readers, this journal provides supporting information supplied by the authors. Such materials are peer reviewed and may be re-organized for online delivery, but are not copy-edited or typeset. Technical support issues arising from supporting information (other than missing files) should be addressed to the authors.
