# Supplementary material for: Electrocatalytic Reduction of Carbon Dioxide to Carbon Monoxide by a Polymerized Film of an Alkynyl-Substituted Rhenium(I) Complex
Source: ChemCatChem. 2013 Apr 12;5(7):1790–6. doi: 10.1002/cctc.201200904 (PMC3738940; doi:10.1002/cctc.201200904)
Supplement: Supplementary file 1 [file cctc0005-1790-SD1.pdf]

Heterogeneous & Homogeneous & Bio-
CHEMCATCHEM
CATALYSIS

Supporting Information

© Copyright Wiley-VCH Verlag GmbH & Co. KGaA, 69451 Weinheim, 2013

Electrocatalytic Reduction of Carbon Dioxide to Carbon Monoxide by a Polymerized Film of an Alkynyl-Substituted Rhenium(I) Complex

Engelbert Portenkirchner,*[a] Jacek Gasiorowski,[a] Kerstin Oppelt,[b] Stefanie Schlager,[a] Clemens Schwarzinger,[c] Helmut Neugebauer,[a] Günther Knör,[b] and Niyazi Serdar Sariciftci[a]

cctc_201200904_sm_miscellaneous_information.pdf

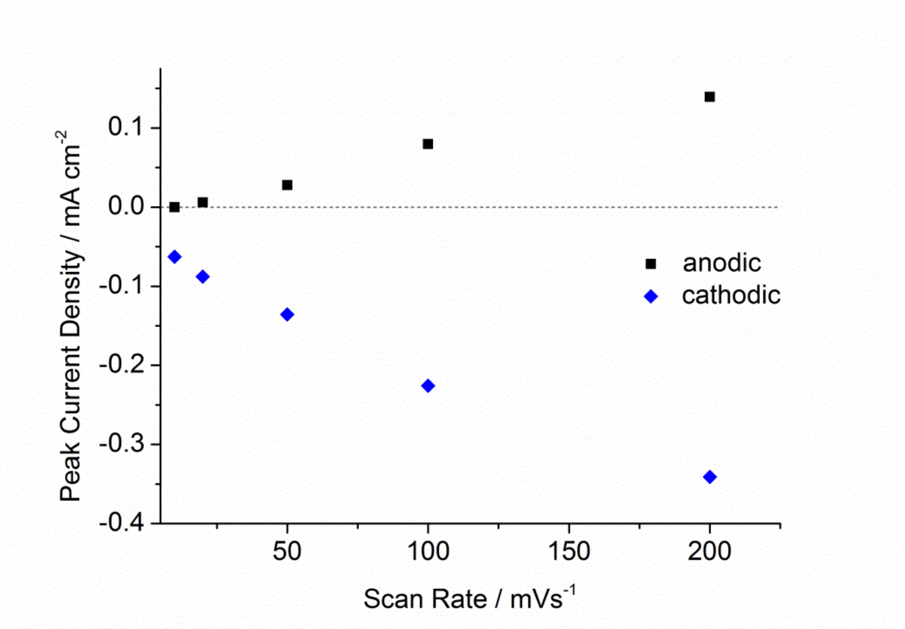

Figure S1. Peak current vs. scan rate dependence according to Figure 2 in the main manuscript. The anodic peak currents were recorded at -1200mV vs. NHE and the cathodic peak currents are recorded at -1600mV vs. NHE. The peak currents depend linearly on the scan rate which is an indication for immobilized redox centers on the electrode surface. [1,2]

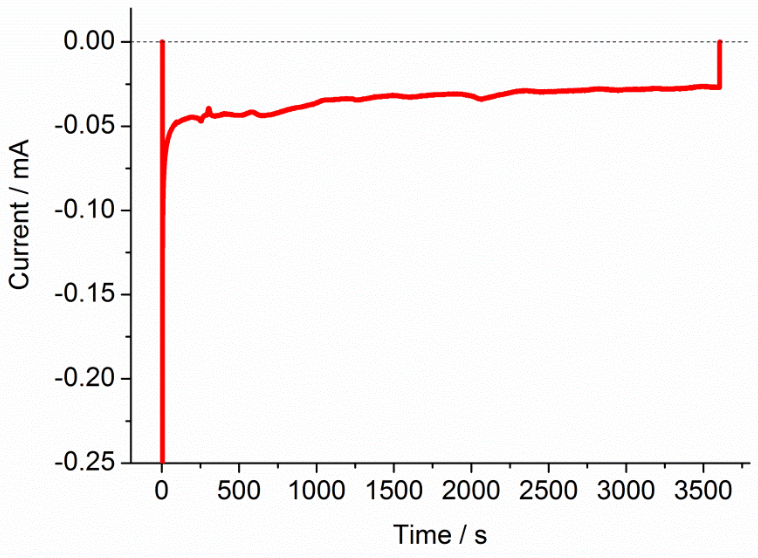

Figure S2. Current vs. time plot for CO_2-electrolysis experiment of the rhenium catalyst film **2** at constant -1600 mV vs. NHE, performed in acetonitrile solution saturated with CO_2 and an electrolysis time of 3600 s. During this time period no film degradation was observed.

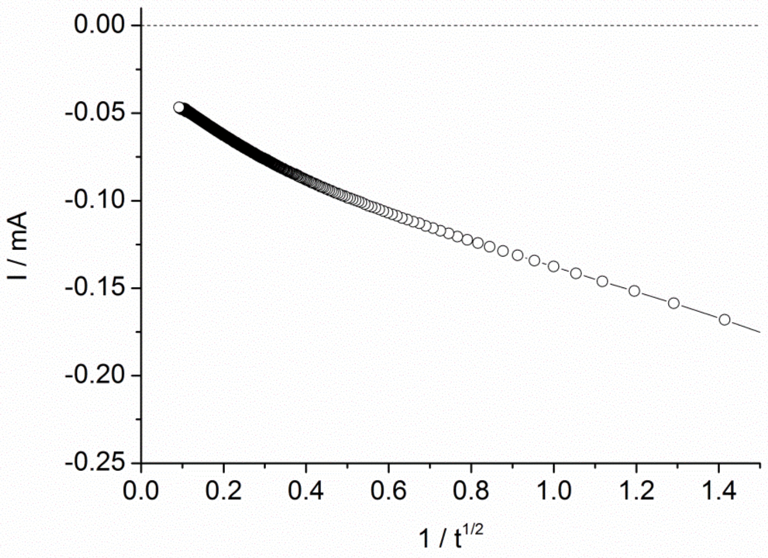

Figure S3. Same data as in figure S2 plotted as current vs. inversed square root of time. According to Fick's second law the linearity of the current vs. $1/t^{1/2}$ plot suggests a fast electron transfer rate and a time-independent surface concentration of the reactant (in this case CO_2) within the electrolysis time of 3600 s.[1]

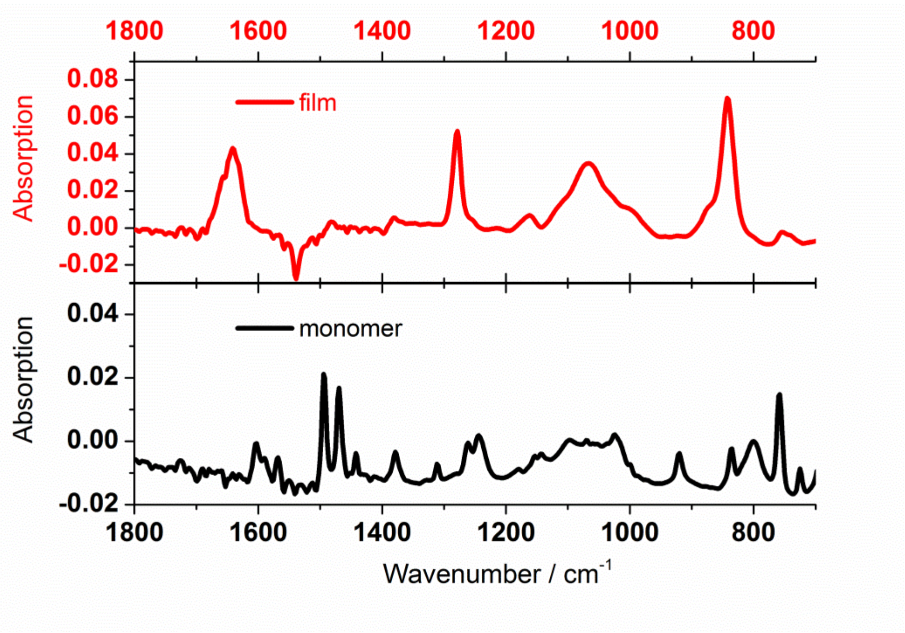

Figure S4. Expansion of the scale from Figure 7 in the main text of the ATR-FTIR difference absorption spectra of a 150nm thick rhenium catalyst film 2 on 10nm Pt sputtered onto a ZnSe ATR crystal to the pure 10nm Pt/ZnSe ATR crystal (top, red solid line) and of the monomer 1 dissolved in DCM and drop cast on a ZnSe ATR crystal to the pure ZnSe ATR crystal (bottom, black solid line)

Reference

[1] A. J. Bard, L. R. Faulkner, *Electrochemical Methods*, John Wiley, New York, **1980**, p171-172, p 218.

[2] J. Heinze, *Angewandte Chemie* **1984**, *11*, 823 – 916.
